# Gold-Doped Hybrid Nanoparticles: A Versatile Tool for Multimodal Imaging of Cell Trafficking

**DOI:** 10.3390/pharmaceutics17121612

**Published:** 2025-12-15

**Authors:** Andrea Bezze, Jessica Ponti, Deborah Stanco, Carlotta Mattioda, Clara Mattu

**Affiliations:** 1 Department of Mechanical and Aerospace Engineering, Politecnico di Torino, Corso Duca Degli Abruzzi 24, 10129 Torino, Italy; 2 European Commission, Joint Research Centre (JRC), 21027 Ispra, Italy

**Keywords:** hybrid nanoparticles, cell trafficking, gold NPs, holotomography, electron microscopy, multimodal imaging

## Abstract

**Background:** Nanomedicine has demonstrated great potential to improve drug delivery across various diseases. However, accurately monitoring the real-time trafficking of organic nanoparticles (NPs) within biological systems remains a significant challenge. Current detection methods rely heavily on fluorescence, while high-resolution, label-free imaging is often precluded by the limited optical contrast of organic materials, limiting a comprehensive understanding of NP fate. Metallic doping allows simultaneous detection of carriers using multiple imaging and analysis techniques. This study presents a novel approach to prepare gold-doped hybrid NPs compatible with multimodal imaging, thus facilitating multimodal tracking. **Methods:** Gold-doped NPs were successfully synthesized via nanoprecipitation, yielding stable, monodisperse carriers with optimal size, confirmed by Dynamic Light Scattering and Nanoparticle Tracking Analysis. UV/Vis spectroscopy confirmed effective gold-doping, with doping efficiency of approximately 50%. Transmission Electron Microscopy (TEM) showed gold NP accumulation throughout the polymer core and near the lipid shell. **Results:** Although gold doping resulted in a slight increase in NP size and zeta potential, no effects on cytocompatibility or cellular uptake by glioblastoma and microglia cells were observed. Furthermore, the optical properties (i.e., the refractive index and the UV spectrum) of the NPs were successfully modified to enable tracking across complementary imaging modalities. Real-time, label-free visualization of NP accumulation in the cytoplasm of U87 cells was achieved via holotomography by exploiting the enhanced refractive index after gold-doping. This observation was confirmed through correlation with fluorescence confocal microscopy, using fluorescently labelled gold-doped NPs. Furthermore, the high electron density of the gold tracer facilitated the precise localization of NPs within intracellular compartments via TEM, bypassing the inherently low contrast of organic NPs. **Conclusions:** These findings validated the gold-doped NPs as versatile nanoplatforms for multimodal imaging, showcasing their potential for non-invasive, high-resolution tracking and more accurate quantification of intracellular accumulation using diverse analytical techniques.

## 1. Introduction

Over the past decades, nanomedicine has emerged as a groundbreaking technology, revolutionising diagnosis and therapy for a broad spectrum of diseases [1,2]. Notably, nanoparticle (NP)-based delivery systems have substantially improved therapeutic outcomes by mitigating off-target toxicity and enhancing drug accumulation in target tissues [3,4]. This enhanced efficacy can be attributed to the inherent advantages of NPs, including improved drug stability, enhanced permeation across biological barriers and increased cell internalisation [5,6]. Several types of NPs, including organic, inorganic, and hybrid platforms, have been proposed [7,8,9]. Among them, polymer and lipid NPs are particularly appealing by virtue of their superior biocompatibility, reduced clearance, and the ability to encapsulate multiple drugs [10,11,12,13]. While lipid-based NPs have historically dominated industrial and therapeutic applications [14], an increasing number of polymer-based nanomedicines have successfully progressed through preclinical and clinical evaluation [1,15]. Indeed, from 2016, polymeric formulations accounted for the largest proportion (29%) of FDA-approved NP-based therapeutics, followed by liposomal (22%) and other lipid-based formulations (21%) [16].

Given the wide spectrum of potential medical applications of polymer NPs, understanding their interactions with living cells is of paramount importance for their full exploitation and clinical translation [17,18]. To this aim, sophisticated imaging and tracking techniques are required to precisely monitor NP localization within cells and intracellular compartments [19,20]. Flow cytometry [21,22] and fluorescence microscopy [23,24] have been extensively employed for this purpose [25]. However, these methods require NP labelling with fluorescent dyes, which may potentially perturb their interaction with biological systems [26,27] and are susceptible to time-dependent photobleaching, resulting in the progressive attenuation of the fluorescent signal [28,29]. Furthermore, the inherent resolution constraints of fluorescence microscopy restrict the detection of individual NPs, typically requiring their aggregation or dense packing for visualisation [25]. Recent advancements in high-resolution microscopy techniques, such as Stimulated Emission Depletion or Laser Scanning microscopy, have enabled the visualisation and tracking of individual NPs, as well as the study of their cell trafficking [30,31]. Similarly, accurate quantification of intracellular localization of fluorescent NPs has been achieved by integrating standard flow cytometry with microfluidics-based tools [32].

However, the widespread adoption of these sophisticated instruments in routine research practice remains limited [33,34]. Recently, metal doping has emerged as a potent tool to study NP distribution in cells and living organisms through more sensitive analyses, such as inductively coupled plasma mass spectrometry (ICP-MS) and transmission electron microscopy (TEM) [32]. TEM is a powerful tool to visualize intracellular compartments and nanoscale structures with high magnification [35], while also enabling qualitative elemental analysis, e.g., through energy dispersive X-ray spectroscopy (EDS). However, the low electron density of polymeric and lipidic NPs, which resembles that of biological tissues, results in poor contrast, limiting their detection through TEM [25]. To enhance the visualization of polymer and lipid NPs under TEM imaging, complex staining procedures, including staining with osmium tetroxide [36] or phosphotungstic acid [37], are needed. Given the complexity and poor selectivity of these staining methods, numerous studies have explored the incorporation of inorganic elements into the particle structure. These electron-dense inorganic components effectively increase contrast, facilitating the identification and tracking of NPs within biological samples [29,38].

Moreover, metal-doped NPs can be visualized using different imaging methodologies that leverage their unique optical properties, enabling fluorescence-free and continuous monitoring inside biological samples [39,40]. For instance, holotomographic microscopy has been proposed for the label-free and real-time localization of metal NPs within living cells, through the detection of variations in the refractive index (RI) between biological samples and metals [41,42]. Indeed, metals exhibit distinct RI values compared to intracellular components, leading to measurable optical phase shifts in the incident light [43,44]. For instance, Kim et al. successfully determined the three-dimensional spatial distribution of gold NPs within live HeLa cells using optical diffraction tomography [45]. As the RI values of polymer NPs and biological tissues are similar, the application of these optical methods to track polymeric nanocarriers remains a challenge.

Recent investigations demonstrated that the incorporation of ultrasmall metallic dopants can effectively modify the RI of polymeric materials [46,47,48], thereby enhancing their differentiation from the surrounding cellular environment [49]. Therefore, in the present work, we explored a novel approach to prepare gold-doped polymer/lipid NPs for multimodal and fluorescence-free imaging. To this aim, we prepared core/shell polymer NPs comprising a polyurethane core and a phospholipid shell and incorporated ultrasmall gold NPs within the polyurethane core and at the core–shell interface.

Previous works by our group have demonstrated that polyurethane (PU) nanoparticles, based on PCL, provide superior versatility, biocompatibility, and encapsulation efficiency, enabling the simultaneous encapsulation of multiple therapeutic and imaging agents [13,50]. The selected phospholipid coating has been demonstrated to significantly enhance particle stability and cytocompatibility both in vitro and in vivo [51,52,53].

Among existing metallic tracers, gold nanoparticles were selected for their established biocompatibility, their previously demonstrated stable encapsulation within the hybrid nanoparticle structure, and potential theranostic applications [54,55,56]. Indeed, gold nanoparticles, if excited with appropriate wavelengths determined by their size and shape, can produce photoacoustic signals acting as ultrasound-based contrast agents and can also generate heat, resulting in photothermal ablation therapy.

Our results confirmed that NPs with the desired structure were obtained and that gold doping did not alter the physicochemical properties and the biocompatibility of the NPs but significantly changed their optical characteristics. Specifically, the refractive index and absorption spectrum were modified in comparison to undoped NPs. As expected, the enhanced refractive index in doped NPs enabled their discrimination from biological structures, allowing real-time detection of NPs trafficking inside cells via holotomography, as well as their identification within cell ultrastructure via TEM imaging. These findings highlight the significant potential of gold doping for high-resolution and real-time label-free evaluation of polymer/lipid NP interactions with cells and intracellular compartments.

## 2. Materials and Methods

### 2.1. Materials

A proprietary poly(ε-caprolactone) (PCL)-based polyurethane (PU, average molecular weight 46,000 Da and a polydispersity of 1.2 [51]) was synthesized through a previously optimized two-step procedure [13].

Briefly, PCL-diol and Hexamethylene diisocyanate (Merk Life Science S.r.l., Milano, Italy) were dissolved in Dichloroethane (Carlo Erba Reagents srl, Cornaredo, Italy) at a 1:2 molar ratio and reacted at 85 °C for 150 min. N-BOC serinol (Merk Life Science S.r.l.) was added in a 1:1 molar ratio to PCL-diol for the chain extension reaction (room temperature, 16 h). The resulting polymer was purified in diethyl-ether/methanol (95:5) and collected by decantation.

L-α-phosphatidylglycerol (Egg, Chicken) (sodium salt) (Egg-PG), 1,2-dioleoyl-sn-glycero-3-phosphoethanolamine-N-[methoxy(polyethylene glycol)-2000] (ammonium salt) (DSPE-PEG), and L-α-Phosphatidylethanolamine-N-(lissamine rhodamine B sulfonyl) Ammonium Salt (Egg-Liss-Rhod PE) were purchased from Avanti Polar Lipids (Merk Life Science S.r.l., Milan, Italy). Gold nanoparticles (5 nm, OD1, suspended in 0.1 mg/mL sodium citrate with stabilizer) were provided by Thermo Scientific Chemicals (Fisher Scientific Italia, Milan, Italy). Analytical grade acetonitrile (ACN) was purchased from Carlo Erba (Carlo Erba Reagents srl, Cornaredo (MI), Italy). Ultrapure water (18.2 MΩ·cm at 25 °C) was obtained using a Milli-Q water station from Merck Millipore. For NP concentration and washing, Amicon^®^ Ultra centrifugal filter units equipped with a 10 kDa molecular weight cutoff (MWCO) membrane were acquired from Millipore^®^ (Merk Life Science S.r.l., Milan, Italy).

### 2.2. Preparation of Gold-Doped NPs

Rhodamine-labelled, gold-doped polymeric NPs (schematic structure reported in Figure S1) were produced via nanoprecipitation, by adapting a previously described procedure [51,53]. Briefly, 50 μL of 5 nm gold NPs (OD1, suspended in 0.1 mg/mL sodium citrate) were added to 300 μL of ACN and stirred at room temperature for 5 min. This suspension was then mixed with 700 μL of a PU solution in ACN (1 mg/mL) at room temperature for 15 min to enable interactions between gold NPs and the polymer. The resulting solution was added drop-wise into 2 mL of water containing 240 μg of Egg-PG, 200 μg of DSPE-PEG, 10 μg of Egg-Liss-Rhod PE, and 5 μg of 5 nm Gold NPs at 60 °C. NPs formed immediately upon precipitation of the polymer/gold NPs mix in water, followed by self-assembly of phospholipids and gold NPs on the outer surface. The obtained NP suspension was collected by centrifugation at 3200 rpm at room temperature, using Amicon^®^ Ultra centrifugal filter units (equipped with a 10 kDa cutoff-membrane), and washed twice with UPW. The NPs were then centrifuged twice at 10,000 rpm for 5 min to pellet the gold-doped NPs and separate them from unbound gold NPs.

### 2.3. Physicochemical Characterization of NPs

The size, polydispersity index (PDI), and surface charge (zeta potential) of gold-doped and undoped NPs were measured through dynamic light scattering (DLS) (Litesizer™ 500, Anton Paar Italia S.r.l., Rivoli, Italy) and assessed daily for 7 days to confirm the stability in water at physiological (37 °C) and storage (4 °C) temperatures. Stability was also evaluated in minimal essential medium (Gibco) supplemented with 10% foetal bovine serum (Gibco) at physiological temperature for 48 h.

DLS analysis was performed through backscatter detection with a scattering angle of 173°. Zeta potential measurements were conducted using Litesizer™ Omega cuvettes (Anton Paar, Italy). All measurements were performed on three independent replicates.

Nanoparticle tracking analysis (NTA) was conducted using the NS500 NanoSight (Malvern Panalytical, Malvern, UK) on diluted samples (1:1000) of undoped and gold-doped NPs. Samples were assessed with a 405 nm laser, and the data were processed by the Nanosight 3.2 software package (NTA build 3.2.16). Undoped NP and gold-doped NP samples were diluted in water to a concentration between 10 and 50 particles per field of view, as required for NTA. A minimum of three recordings (60 s) were captured using an EM-CCD camera and employed to determine the NP size, density, and number-based distribution.

The optical properties of both gold-doped and undoped NPs were assessed by measuring the refractive index (RI) of the NP suspension and by determining their absorption spectrum via UV-Vis spectrophotometry. RI measurements at a wavelength of 658 nm were conducted for 5 nm gold NPs, undoped NPs, and gold-doped NPs, suspended in 1 mL of water. Measurements taken across three independent samples were performed using a Litesizer™ 500 instrument (Anton Paar) at 25 °C.

Absorption spectra were recorded using a Varioskan™ LUX Multimode Microplate Reader (Thermo Fisher Scientific Inc., Waltham, MA, USA) with samples housed in 96-well plates. Spectral acquisition was performed across a wavelength range of 200–800 nm, with data points collected at 2 nm intervals. Instrument operation and data retrieval were managed using the SkanIt RE software (version 6.0.1, Thermo Fisher Scientific Inc., Waltham, MA, USA). To assess the spectral contribution specifically associated with the incorporated gold NPs, the absorption spectrum of the undoped polymer NPs was subtracted from the spectrum of the gold-doped NPs.

Direct and indirect methods were used to determine 5 nm gold NP loading inside the hybrid NPs [57]. Quantification of gold loading efficiency within the doped NPs was achieved indirectly by determining the concentration of unencapsulated gold NPs in the supernatant collected post-nanoprecipitation. Unencapsulated gold NPs were quantified by detecting their characteristic absorbance at 518 nm through the Varioskan™ LUX Multimode Microplate Reader. The loading efficiency (*LE*) of gold NPs inside the polymeric particles was determined through Equation (1):

(1) $$LE_{\text{indirect}}\left(\%\right)=\frac{\text{Total}\;\text{Gold}\;\text{NPs}\;-\;\text{free}\;\text{Gold}\;\text{NPs}}{\text{Total}\;\text{Gold}\;\text{NPs}}\times 100$$

where *Total Gold NPs* is the amount of 5 nm gold NPs initially supplied during the synthesis, and *free Gold NPs* is the amount of unencapsulated 5 nm gold NPs measured by UV spectroscopy.

Additionally, gold *LE* was also estimated through a direct method on the collected NPs by measuring their characteristic absorbance at 518 nm through the Varioskan™ LUX Multimode Microplate Reader after removing the background associated with the other components of the hybrid particles. The *LE* of gold NPs inside the polymeric particles was then determined through Equation (2):

(2) $$LE_{\text{direct}}\left(\%\right)=\frac{\text{Encapsulated}\;\text{Gold}\;\text{NPs}}{\text{Total}\;\text{Au}\;\text{NPs}}\times 100$$

where *Encapsulated Gold NPs* is the amount of encapsulated 5 nm gold NPs measured by UV spectroscopy.

### 2.4. In Vitro Characterization of NPs

In vitro toxicity of undoped and gold-doped NPs was evaluated on Human glioblastoma cells (U87-MG, ATCC^®^ HTB14TM) and human microglia cells (HMC3, ATCC^®^ CRL3304TM). Cells were cultured in a humidified incubator (37 °C, 5% CO_2_, and 95% humidity), in Gibco minimal essential medium (MEM) supplemented with 10% (U87-MG) or 12% (HMC3) foetal bovine serum and 1% penicillin/streptomycin (10,000 U/mL) (all reagents from Gibco, Rome, Italy). Human astrocyte cells (HASTR/ci35, conditionally immortalized, clone 35) were kindly provided by Professor Tomomi Furihata, Tokyo University of Pharmacy and Life Sciences (Japan). Cells were routinely maintained in Gibco™ Astrocyte Medium, supplemented with 1% P/S and 4 μg/mL Blasticidin S Hydrochloride (Fisher BioReagents, Fisher Scientific Italia, Milan, Italy), in T75 flasks coated with collagen-I (5 μg/cm^2^, Corning^®^, New York, NY, USA).

For toxicity evaluation, cells (10,000 cells per well) were cultured in 96-well plates for 24 h, followed by incubation with gold-doped NPs at different concentrations (50, 100, 200, 500 μg/mL). Cell viability was assessed at 24 h, 48 h, and 72 h through CellTiter 96^®^ AQueous One Solution Cell Proliferation Assay (Promega Italia S.r.l., Milan, Italy). Cell viability was reported as the percentage of viable cells relative to untreated cells at the same time points.

For the quantification of NP internalisation, U87-MG, HMC3 and HASTR were plated in 24-well plates (100,000 cells/well) and cultured for 24 h in complete medium. The medium was then substituted with 500 µL of rhodamine-labelled gold-doped NPs suspension in complete medium at different concentrations (100, 200, and 500 μg/mL) and incubated for 2 h and 48 h. After incubation, cells were washed twice with sterile phosphate-buffer saline (PBS, Gibco, Rome, Italy) and detached using trypsin-EDTA (0.25%, Gibco, Rome, Italy). The collected cells were centrifuged and washed with sterile PBS. Untreated cells (i.e., cells incubated with fresh culture media) were used as controls. Cells were analyzed by Guava EasyCyte TM 6-2L (Luminex Corporation, Austin, TX, USA) flow cytometer combined with GuavaSoft 3.2 software. Data were collected from 5000 events per sample (*n* = 4).

NP uptake was qualitatively assessed through confocal microscopy. Cells were seeded at a density of 100,000 cells/well in 24-well plates containing circular cover slips. The cells were cultured for 24 h before being exposed to rhodamine-labelled nanoparticles at concentrations of 200 and 500 μg/mL. Following a 24-h incubation period, the cells were prepared for confocal microscopy. For staining, cells were rinsed twice with 500 μL of PBS and fixed with 250 μL of 4% paraformaldehyde in PBS (Fisher Scientific Italia, Milan, Italy) for 20 min. After two subsequent washing steps with PBS, 250 μL of Blocking Buffer (SuperBlock™, Fisher Scientific Italia, Milan, Italy) was added for 30 min. Nuclei and F-actin filaments were then counterstained with 4′,6-diamidino-2-phenylindole, dihydrochloride (DAPI, Invitrogen, Life Technologies Europe BV, Milano San Felice, Italy) and Alexa Fluor^®^ 488 Phalloidin (Cell Signaling Technology^®^, Leiden, The Netherlands), according to the manufacturer’s instructions. Finally, images of the cell cytoplasm, nuclei, and nanoparticles were captured using a Nikon ECLIPSE Ti2 inverted confocal microscope. Images were processed using NIS-Elements Advanced Research software (Nikon) and Fiji software (version 1.54p, National Institutes of Health, Bethesda, MD, USA).

### 2.5. Fluorescence-Free High-Resolution Imaging

Interactions of gold-doped nanoparticles were assessed using fluorescence-free high-resolution imaging techniques that rely exclusively on the intrinsic contrast properties of gold nanoparticles, i.e., holotomography and TEM.

To assess NP internalization and distribution inside cells through holotomography, U87-MG cells were cultured in black 6-well glass-bottom plates (Cellvis, Mountain View, CA, USA) for 24 h and then incubated with different concentrations of rhodamine-labelled gold-doped NPs (100, 200, and 500 μg/mL). 3D Refractive Index (RI) distribution and fluorescence images were acquired over 12 h using a holotomographic microscope (Tomocube HT-X1™; Tomocube Inc., Daejeon, Republic of Korea). Images were reconstructed and processed through TomoStudio™ software (TomoAnalysis V2.x, Tomocube Inc., Daejeon, Republic of Korea) and Fiji software (version 1.54p, Nation-al Institutes of Health, Bethesda, MD, USA). Average RI histograms were generated from cellular RI tomograms using MATLAB R2024a (MathWorks, Natick, MA, USA). To accurately delineate cellular boundaries from background noise, regions exhibiting RI values below 1.34 were excluded from the analysis. Quantitative analysis of NP distribution inside cells was performed on reconstructed 3D tomograms using ImageJ (version 1.54p) for image processing and GraphPad Prism (GraphPad Software, San Diego, CA, USA) for statistical evaluation. Pearson’s correlation coefficient (r) was calculated to assess the linear relationship between variables. Statistical significance was evaluated with a two-tailed test (α = 0.05).

For TEM, undoped and gold-doped NPs were fixed using 4% osmium solution (Sigma Aldrich, Milan, Italy). A 3 µL drop from the NP suspension was manually deposited on Formvar carbon-coated 200 mesh copper grids (Agar Scientific, London, UK), left to dry, and stained with Uranyl acetate for 5 min. Samples were imaged with a JEOL JEM-2100 HR-transmission electron microscope at 120 kV (JEOL, Basiglio, Italy) coupled with an X-Flash Detector 5030 (Bruker, Milano, Italy). The size distribution was manually calculated on at least 450 particles from TEM images by using ImageJ software (version 1.54p).

The NP interaction with cell membrane and the uptake of gold-doped NPs inside U87-MG cells were evaluated by TEM. U87-MG cells were seeded in 10 cm Petri dishes, in 5 mL of culture medium, with a final seeding density of 200,000 cells/mL. Cells were allowed to adhere for 24 h and exposed for 24 h to gold-doped NPs at a concentration of 500 μg/mL. Unexposed cells were used as controls. After incubation with NPs, cells were washed twice with PBS, harvested, re-suspended in a 2% Karnovsky solution containing paraformaldehyde and glutaraldehyde, and fixed in osmium tetroxide solution with 0.1 M cacodylate at pH 7.3 for 1 h. After three washes in 0.05 M cacodylate (10 min each), cells were dehydrated with serial ethanol solutions in water (30%; 50%; 75%; 95% for 15 min, and 100% for 30 min), then incubated in absolute propylene oxide for 20 min and embedded in a 1:1 solution of epoxy resin/propylene oxide for 90 min. This mixture was renewed with pure epoxy resin and maintained overnight at room temperature, followed by polymerization at 60 °C for 48 h. Ultrathin sections (50–70 nm) were obtained using a Leica EM UC7 ultramicrotome (Leica, Milan, Italy) and stained for 2 min with uranyl-less solution (TAAB Laboratories Equipment Ltd., Aldermaston, UK) and lead citrate Reynolds solution (TAAB Laboratories Equipment Ltd., UK) for 2 min, then washed and dried. Ultrathin sections were collected on Formvar carbon-coated 200 mesh copper grids (Agar Scientific, London, UK) and imaged with a JEOL JEM-2100 HR-transmission electron microscope at 120 kV (JEOL, Milan, Italy). All chemicals, unless otherwise stated, were purchased from Sigma Aldrich (Milan, Italy).

### 2.6. Statistical Analysis

All results are presented as mean ± standard deviation (SD). The specific sample size (*n*) for each experimental condition is reported in the figure captions. T-Test analysis with a 95% confidence interval was employed for comparisons between the gold-doped NP and the undoped NP groups. Multiple comparisons were performed through one-way ANOVA with Tukey correction with a confidence interval of 95%. Statistical analysis was performed with GraphPad 9 Prism software (GraphPad, San Diego, CA, USA). The statistical significance was defined as follows: * *p* < 0.05 (statistically significant), ** *p* < 0.01 (highly significant), *** *p* < 0.001 (very highly significant), and **** *p* < 0.0001 (extremely significant). Statistical analysis and data visualization were performed using GraphPad Prism 9 (GraphPad Software, San Diego, CA, USA).

## 3. Results

### 3.1. Impact of Gold-Doping on the Physicochemical Properties of NPs

**Figure 1:**
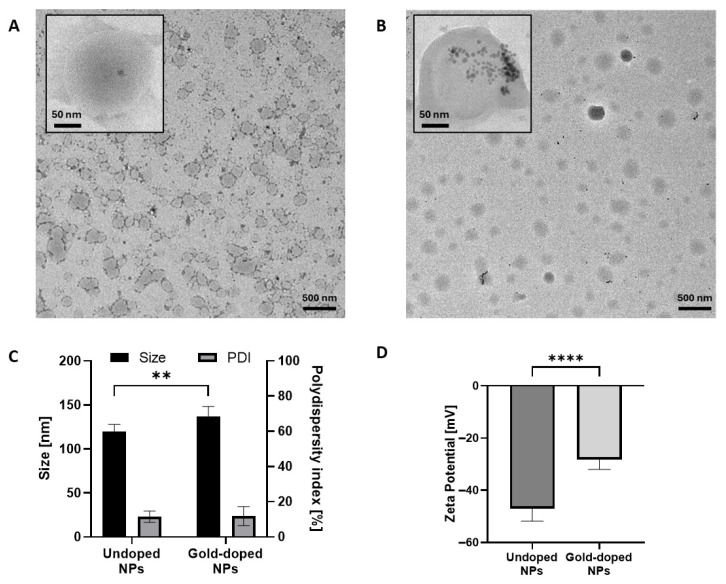
TEM images of undoped (**A**) and gold-doped (**B**) NPs. Insets show high-magnification details of individual NPs. Hydrodynamic diameter and polydispersity index (PDI) (**C**) and Z-potential (**D**) of undoped NPs and gold-doped NPs (*n* = 6). Statistical analysis was by Ordinary Two-way ANOVA (size, PDI) or unpaired t-test (Zeta potential), comparing the two groups. ** *p* < 0.01, **** *p* < 0.0001.

Undoped and gold-doped hybrid polymer/lipid NPs with the desired structure were successfully obtained through nanoprecipitation, as confirmed by TEM images (Figure 1A and B, respectively). Particles exhibited a spherical morphology and the desired core–shell structure. High magnification TEM micrographs showed distinct dark spots in the gold-doped NPs corresponding to the high electron-dense 5 nm gold NPs (inset of Figure 1B), which were absent in the undoped NPs (inset of Figure 1A), confirming the successful incorporation of the metallic dopant. Gold NPs were distributed throughout the entire particle volume, both in the polymer core and near the lipid shell, with some agglomeration. The particle density detected by NTA (Table 1) confirmed that a significant amount of undoped and gold-doped NPs were produced, obtaining a concentration of 3.3 ± 0.1 × 10^12^ and 4.0 ± 0.2 × 10^12^ particles/mL, in line with previous reports on NPs produced through nanoprecipitation [58,59].

The presence of gold inside the doped NPs was further quantified by UV/Vis spectroscopy, obtaining a gold doping efficiency of 56.3 ± 7.6% with indirect measurement (i.e., by detecting the amount of gold NPs remaining in the supernatant) and of 47.7 ± 2.6% by direct measurement of gold inside the NPs (Table 1). Both measurements were coherent (*p* = 0.2059) and in line with other studies on the encapsulation of ultrasmall gold NPs inside micro- and nano-carriers [60,61,62].

Both types of NPs displayed a small size, with a diameter within the 100–200 nm range and a narrow size distribution (Figure 1C, Table 1). The hydrodynamic diameter, measured by DLS, slightly increased upon doping with gold, from 120 ± 8 nm for undoped NP to 140 ± 13 nm for gold-doped NPs. The size distribution histograms derived from manual measurements of particle diameter from TEM images (Figure S2A,B) as well as NTA results (Table 1) were consistent with DLS measurements and confirmed the narrow size distribution for both NP types. Gaussian fitting from TEM images revealed a mean diameter of 124 ± 43 nm for undoped NPs and 141 ± 53 nm for gold-doped NPs (Figure S2A,B, Table 1), while NTA revealed an increase in particle size from 101 ± 2 nm to 124 ± 1 nm (Table 1). Both undoped and gold-doped NPs maintained a low PDI (Figure 1C) of 12 ± 3% and 10 ± 6%, respectively, with negligible differences, as also reported in previous studies incorporating metallic dopants in NPs [9,63,64,65]. Both undoped NPs and gold-doped NPs exhibited a net negative surface charge (Figure 1D), which is expected to guarantee their stability in suspension [66,67]. As expected, the introduction of gold NPs, which possess a near-neutral surface charge (Table S1), produced a statistically significant decrease in the absolute value of the zeta potential for the gold-doped NPs, from −47 ± 5 mV to −28 ± 4 mV.

**Figure 2:**
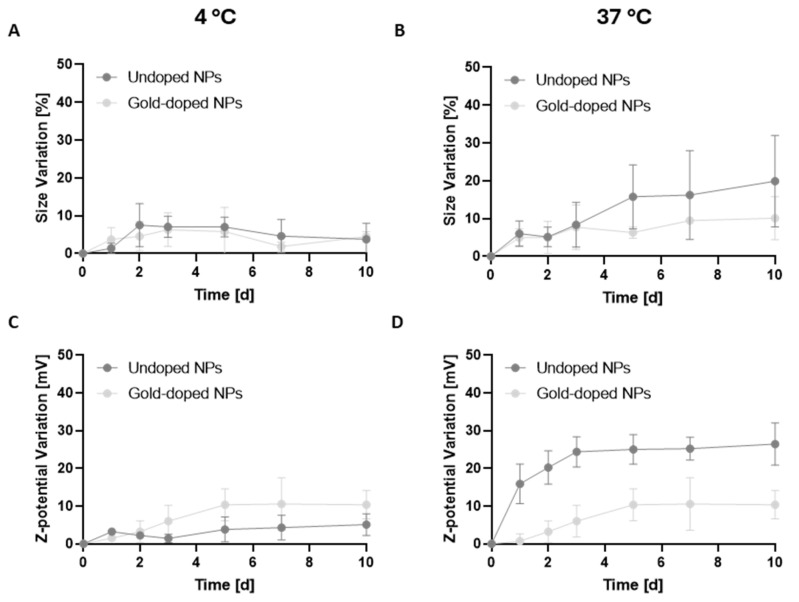
Mean variation (%) of the hydrodynamic diameters for undoped and gold-doped NPs over time, after incubation in ultra-pure water at 4 °C (**A**) and 37 °C (**B**) (*n* = 3). Mean variation in the z-potential for undoped and gold-doped NPs over time, after incubation in ultra-pure water at 4 °C (**C**) and 37 °C (**D**) (*n* = 3).

Gold-doped NPs demonstrated good long-term stability under storage (4 °C) and physiological (37 °C) temperatures. No significant variations in size (Figure 2A,B) or PDI (Figure S3) were observed over 10 days of incubation in water, with a similar behaviour between the two types of NPs. The average variation in the hydrodynamic diameter under storage conditions over time remained below 10% for both NP types, while a more pronounced size increase was observed under physiological conditions after 10 days. The average size variation was lower for gold-doped NPs in comparison to undoped particles, but no statistically significant differences were observed, confirming a similar behaviour for the two formulations. Surface charge variation detected for gold-doped NPs was significantly lower than the variation observed for the undoped NPs. Indeed, at 4 °C (Figure 2C), the zeta potential value of the gold-doped NPs ranged from −27 ± 4.7 mV to −23 ± 0.9 mV after 10 days, while the undoped NPs displayed a significant change in the zeta potential, which varied from −49 ± 5.3 mV to −26 ± 0.8 mV, with a variation of 23 mV. A similar behaviour was observed after incubation at 37 °C (Figure 2D). The surface charge changed from −29 ± 3.4 mV to −19 ± 0.4 mV for gold-doped NPs, and from −46 ± 4.6 mV to −19 ± 1.4 mV for the undoped ones. These results suggest that the gold-doping process did not hinder the physicochemical stability of the NPs and seemed to stabilize the surface charge over time.

The colloidal stability of the nanoparticles in serum-supplemented culture medium at physiological temperature was also assessed. The gold-doped NPs remained stable during the initial 6 h of incubation, followed by a gradual increase in hydrodynamic size (Figure S4A). A similar trend was observed for undoped NPs, further confirming that the incorporation of Au NPs did not affect colloidal stability. Furthermore, the observed increase in size was slower and less pronounced than the values reported for other polymer-based particles, confirming the effective mitigation of protein adsorption by the phospholipid shell [68,69]. Moreover, both NP types maintained PDI values below 20%, compatible with a monodisperse suspension, despite a slight and gradual increase throughout the incubation period (Figure S4B). Surface charge variations detected for the gold-doped NPs (shifting from −27 ± 4 mV to −21 ± 1 mV) were significantly less pronounced than observed for the undoped NPs (from −51 ± 2 mV to −20 ± 3 mV (Figure S4C), indicating that the gold-doping process did not hinder the physicochemical stability of the nanoparticles. Conversely, the process appeared to contribute to a more stable surface charge over time, even under incubation in the more biologically relevant cell culture medium.

### 3.2. Impact of Gold-Doping on the Biological Properties of NPs

**Figure 3:**
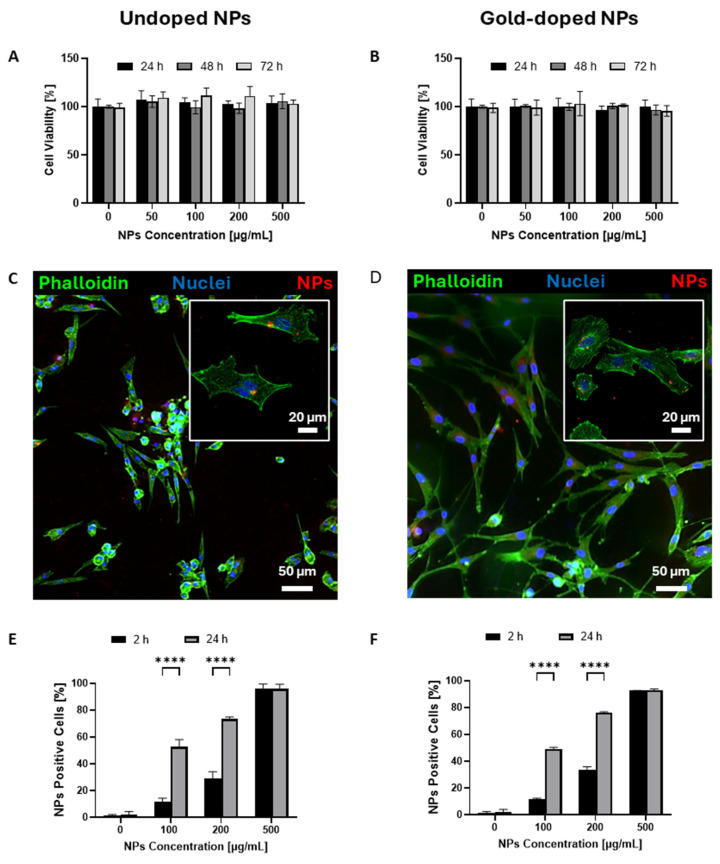
(**A**,**B**) Cell viability assay after incubation of U87-MG cells with undoped (**A**) and gold-doped (**B**) NPs at different concentrations for 24 h, 48 h, and 72 h (*n* = 3). (**C**,**D**) Representative confocal imaging of rhodamine-labelled undoped (**C**) or gold-doped (**D**) NPs internalized by U87-MG after 24 h of incubation with a concentration of 500 μg/mL of NPs. F-actin was stained with phalloidin (green), nuclei were stained with DAPI (blue), and NPs were visualized in red. Insets on the upper right show high-magnification images of cells. (**E**,**F**) Flow cytometry quantification of the percentage of NP-positive cells on U87-MG cells after 2 and 24 h incubation with different concentrations of rhodamine-labelled undoped (**E**) or gold-doped (**F**) NPs (*n* = 4). **** *p* < 0.0001. Statistical analysis was performed by Ordinary Two-way ANOVA, comparing the groups treated with the same concentrations at the two timepoints.

Regardless of concentration and incubation time, no signs of cytotoxicity were observed on U87-MG cells after exposure to undoped (Figure 3A) and gold-doped (Figure 3B) NPs for up to 72 h, confirming that the addition of the metallic dopant did not alter the biocompatibility of the hybrid formulation. Additionally, gold-doped NPs were also well tolerated by normal resident brain cells, i.e., human microglia and astrocytes, as shown in Supplementary Figure S5A and B, respectively.

Confocal microscopy images (Figure 3C,D) confirmed NP uptake by U87-MG cells for both undoped and gold-doped NPs, as the fluorescent signal associated with rhodamine-labelled NPs was detected in the cell cytoplasm, with no significant differences between the two conditions. Gold-doped NPs were also successfully internalized by normal brain microglia (Figure S5A) and human astrocytes (Figure S7A), with a similar internalization trend as observed for U87-MG tumour cells (Figure S6B,C). However, the percentage of NP-positive microglia cells was significantly lower compared to U87-MG cells under the same conditions (Figures S6D and S7B).

Flow cytometry analysis on U87-MG (Figure 3E,F) confirmed high cell internalization for both undoped and gold-doped NPs by U87-MG cells. As expected, the internalization increased with the concentration and the incubation time, in line with literature data [68]. No significant differences were observed between the two types of NPs, suggesting that the gold-doping process did not alter the cellular uptake kinetics. NP internalization increased over time for all concentrations, suggesting a progressive dynamic accumulation of NPs within U87-MG cells (Figure S8A,B). For low NP concentrations, the number of NP-positive cells increased over time, reaching more than 50% internalization after 24 h.

**Figure 4:**
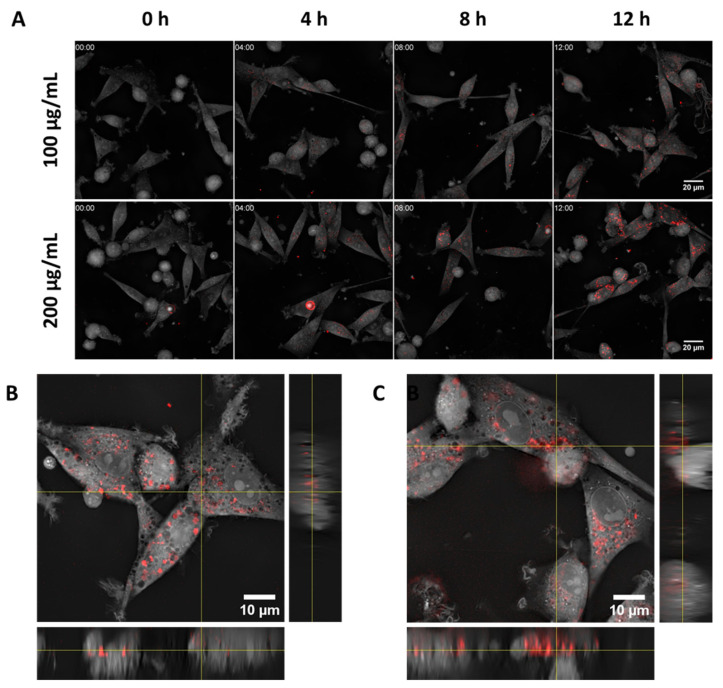
(**A**) Internalization kinetics of gold-doped NPs (100 μg/mL and 200 μg/mL) by U87-MG cells at selected timepoints (0, 4, 8, 12 h) shown by combining fluorescence microscopy and holotomography imaging. Rhodamine-labelled gold-doped NPs are shown in red over grayscale holotomography images. (**B**,**C**) Z-stack images of U87-MG cells incubated for 12 h with rhodamine-labelled gold-doped NPs at 100 μg/mL (**B**) and 200 μg/mL (**C**).

At the highest concentration tested (500 μg/mL), over 90% of the cells had internalized NPs after 2 h, demonstrating the rapid uptake by tumour cells, with no significant changes observed after 24 h. Notably, while the percentage of NP-positive cells remained stable between 2 and 24 h, the mean fluorescence intensity (Figure S8C) doubled, suggesting a sustained internalization process beyond the initial uptake phase [69,70]. A similar internalization dynamic was also observed for astrocytes (Figure S7C), while no significant mean fluorescent intensity increase over time was detected for microglia (Figure S6E). Live microscopy images confirmed a time- and concentration-dependent internalization by U87-MG cells (Figure 4A and Figure S9). The internalization process was rapid, with evident intracellular accumulation of NPs after 40 min for the highest NP concentration of 200 μg/mL, likely facilitated by the affinity between the phospholipid shell of the NPs and the cell membrane [71,72]. Furthermore, representative Z-stack images (Figure 4B,C) confirmed the predominant localization of NPs within the cell cytoplasm, demonstrating successful translocation across the plasma membrane.

### 3.3. Effect of Gold Doping on the Optical Properties of NPs

**Figure 5:**
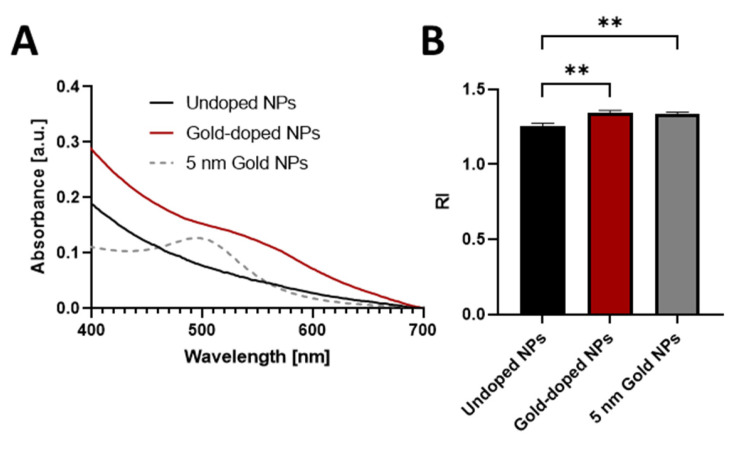
(**A**) UV-Vi’s absorption spectra of undoped NPs (black line), gold-doped NPs (red line), and 5 nm gold NPs (dotted line) in UPW. (**B**) Refractive index (RI) of undoped NPs, gold-doped NPs, and 5 nm gold NPs in UPW (*n* = 3). Statistical analysis was by Ordinary One-way ANOVA, comparing the three groups with each other. ** *p* < 0.01.

UV-Vis spectrophotometric analysis showed a distinctive surface plasmon resonance (SPR) peak at 518 nm for bare gold NPs (Figure S10A), with a near-linear correlation between the absorbance and the concentration of gold NPs (Figure S10B). The absorption spectra of undoped and gold-doped NPs (Figure 5A) clearly show a new peak at 518 nm for the gold-doped NPs, which was absent in the undoped ones, confirming successful doping. In the gold-doped NP spectrum, a pronounced broadening of the SPR peak and a discernible shift towards longer wavelengths (redshift) were observed in comparison to bare gold NPs [73,74,75]. This phenomenon, extensively documented in the literature, is commonly attributed to the aggregation of gold NPs into agglomerates of varying dimensions within the polymer matrix [76,77,78].

The successful integration of gold NPs was also demonstrated by a discernible increase in the RI of the gold-doped NPs (Figure 5B), compared to undoped ones [79,80]. The RI of the undoped NPs (1.257 ± 0.018) was comparable to the reported RI of the cell cytoplasm [81], while gold-doped NPs presented a higher RI (1.343 ± 0.016, Table 1), comparable to the RI of free gold NP (1.336 ± 0.012) [45]. Overall, the incorporation of the gold-dopant produced the desired modification of the optical properties of NPs, which can be exploited for fluorescence-free imaging.

### 3.4. Fluorescence-Free High-Resolution Imaging of Cell Trafficking of Gold-Doped NPs

**Figure 6:**
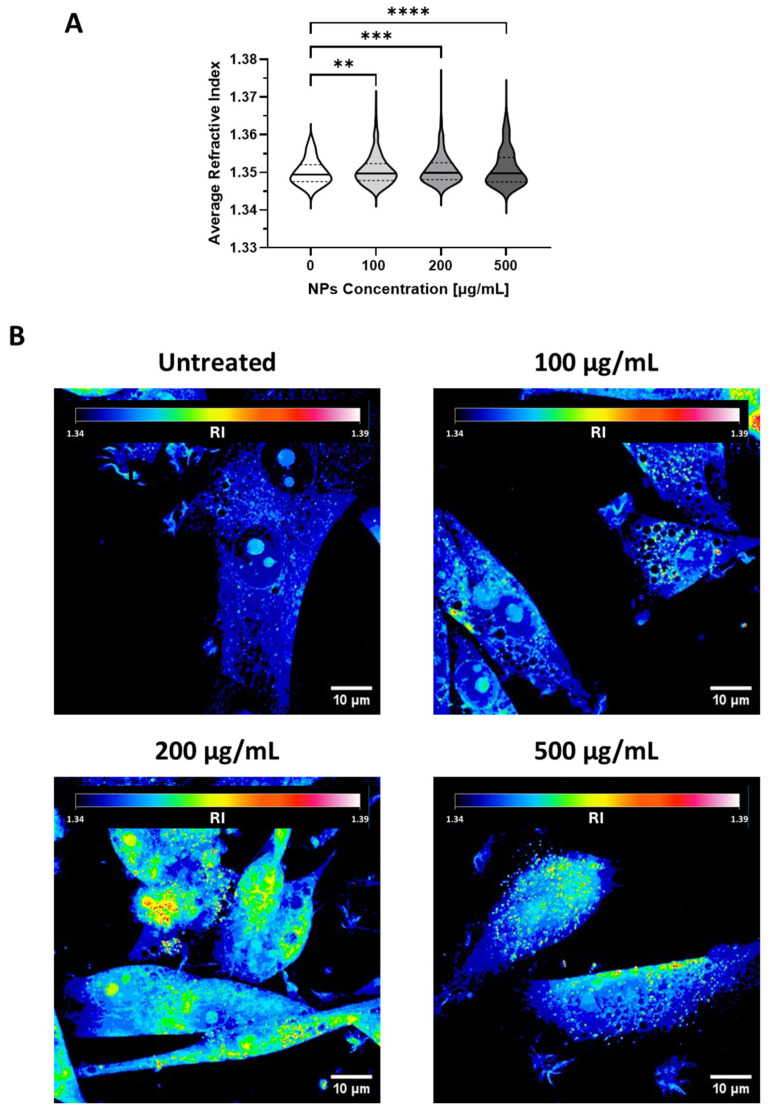
(**A**) Violin plot of the average RI of U87-MG cells incubated with different concentrations of gold-doped NPs. The mean values are reported with a continuous line, while the dotted lines represent the first and third quartiles. Data were collected on holotomography images of at least 700 cells. Statistical analysis was by Ordinary One-way ANOVA, comparing each group with the control group. ** *p* < 0.01, *** *p* < 0.001, **** *p* < 0.0001. (**B**) Representative RI holotomography images of native U87-MG cells and cells incubated with gold-doped NPs at different concentrations: 100 μg/mL, 200 μg/mL, 500 μg/mL.

Previous literature suggests that holotomography can be potentially employed to assess the distributions of gold-doped NPs within cells, as an alternative to fluorescent label-based confocal microscopy [45,49]. We acquired three-dimensional RI tomograms of U87-MG cells incubated with increasing concentrations of gold-doped NPs. We found that the average RI was significantly altered upon exposure to gold-doped NPs (Figure 6A). Specifically, cells incubated with increasing concentrations of gold-doped NPs displayed a significant increase in the number of pixels with RI exceeding 1.36, compared to untreated control cells (Figure S11A–D), confirming that gold-doped NPs can be employed in the label-free monitoring of their intracellular accumulation [45]. The representative holographic image in Figure 6B revealed that native U87 cells possess a largely homogeneous RI distribution across the cytoplasm, with only limited local variations corresponding to intracellular organelles. The incubation with gold-doped NPs did not induce observable alterations in the cell morphology. However, distinct domains associated with high RI values appeared within the cell cytoplasm after treatment with gold-doped NPs (Figure 6B, 100 μg/mL). Both extension and RI intensity of these domains increased proportionally with the concentration of gold-doped NPs (Figure 6B, 200 and 500 μg/mL). Previous studies, albeit conducted with different cell lines and other metallic particles [45,82], identified these high-RI volumes as NP agglomerates confined within intracellular compartments, such as the endosomes or the lysosomes.

**Figure 7:**
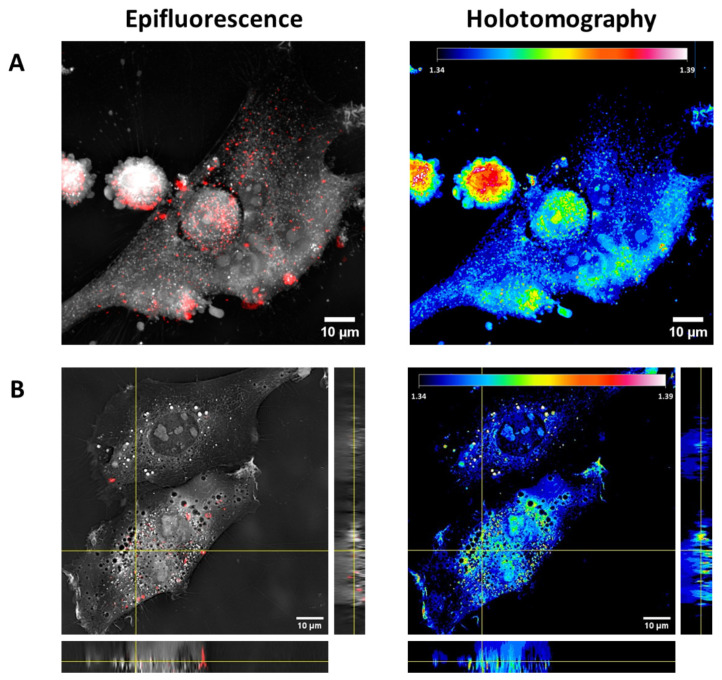
Epifluorescence and holotomography images of U87-MG cells incubated for 2 h with rhodamine-labelled gold-doped NPs (500 μg/mL). In epifluorescence images, rhodamine-labelled gold-doped NPs are shown in red over the grayscale holotomography reconstruction. (**A**) Representative maximum intensity projection images. (**B**) Representative Z-stack projections.

To confirm the presence of gold-doped NPs in the regions with high RI, holotomography and confocal fluorescence images were simultaneously acquired (Figure 7). Representative maximum projection images of cells treated with gold-doped NPs labelled with rhodamine demonstrate a strong spatial correlation between regions of high RI and the fluorescent signals of NPs (Figure 7A). Quantitative analysis confirmed that 92 ± 3% of the fluorescent signal from NP was colocalized with the high-RI voxels (Table 2), confirming that RI measurements with holotomography can detect gold-doped particles. Some non-overlapping regions were observed as previously documented by other studies [45,82,83], which may be attributed to the presence of high-density intracellular structures such as lipid droplets [84,85], which present RI values of 1.37–1.49 [86,87,88] comparable to those of gold-doped NP aggregates. However, these structures constituted a minor fraction of the total cell volume in both treated and untreated samples (Figure 6B), thus minimising the occurrence of false-positive gold-doped NP detection based on RI signal. Three-dimensional images (Figure 7B) clearly demonstrate that the gold dopant acted as an RI-based contrast agent, enabling the monitoring of NP internalisation and three-dimensional intracellular distribution via holotomography. Furthermore, holotomography facilitated the simultaneous, label-free visualisation of endogenous cellular components and allowed the simultaneous tracking and intracellular localisation of gold-doped NPs, with a resolution comparable to confocal microscopy.

**Figure 8:**
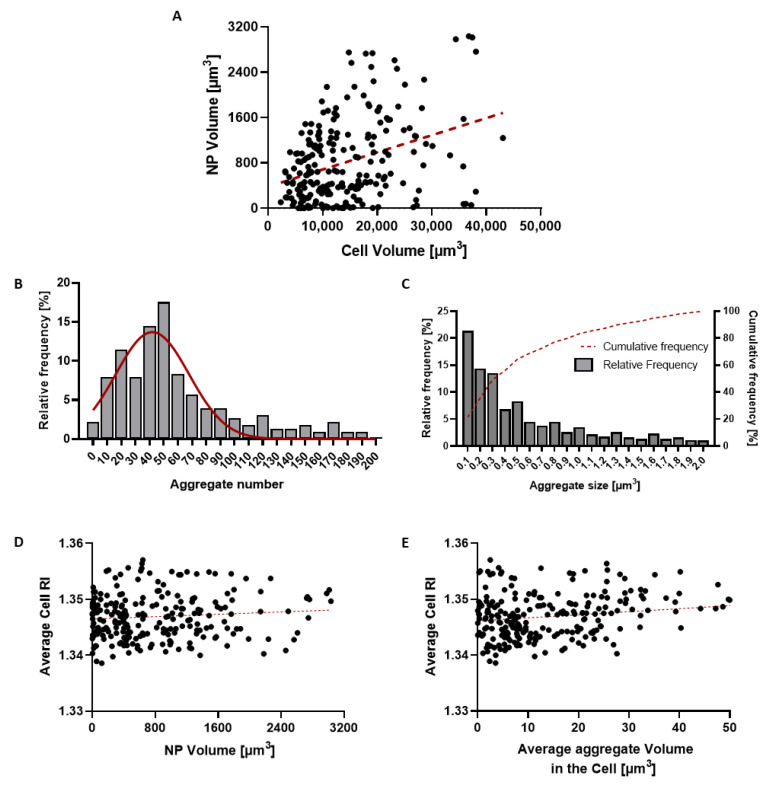
Quantitative analysis of NP distribution within cells. (**A**) Scatter plot showing the relationship between cell volume and the volume of NP aggregates, suggesting a significant positive association (r = 0.3534; *p* < 0.0001). (**B**) Distribution of the number of NP aggregates per cell with gaussian fit curve (in red). (**C**) Aggregate size distribution with cumulative frequency (in red). (**D**) Scatter plot showing the relationship between total NP volume per cell and average cellular RI, indicating no significant correlation (r = 0.0942; *p* = 0.155). (**E**) Scatter plot showing the relationship between the average size of NP aggregates and cellular RI, revealing a modest positive correlation (r = 0.2148; *p* = 0.0009).

Quantitative analysis of 3D tomograms was performed to evaluate the accumulation and distribution of NP within cells. Figure 8A illustrates the relationship between the volume of high-RI regions within the cell (corresponding to gold-doped NP aggregates) and the volume of the cell. On average, NP aggregates accounted for 6.7 ± 5.5% of the cell volume (Table 2). Correlation analysis revealed a weak but statistically significant positive association (r = 0.3534; *p* < 0.0001) between cell volume and the volume occupied by NPs inside the cell, with smaller cells showing lower NP accumulation, consistent with previous evidence [89].

The number of NP aggregates per cell followed an approximately Gaussian distribution (Figure 8B), with a mean of 50 aggregates (Table 2). The average volume of the aggregates was 0.55 ± 0.50 µm^3^ (Table 2). The aggregate size followed a non-Gaussian distribution, with high-volume aggregates being less frequent (Figure 8C). Notably, over 80% of the measured aggregates showed a size lower than 1 µm^3^, consistent with confinement within intracellular endosomes [90].

The potential effect of NP accumulation on the RI of the cells was also assessed. No significant correlation was observed between the total volume of NPs per cell and the mean cell RI (Figure 8D; r = 0.0942; *p* = 0.155). However, a significant association was found between the mean cell RI and the average size of NP aggregates (Figure 8E; r = 0.2148; *p* = 0.0009), suggesting that cell RI increase is driven more by NP clustering within intracellular compartments than by the amount of internalized NPs.

**Figure 9:**
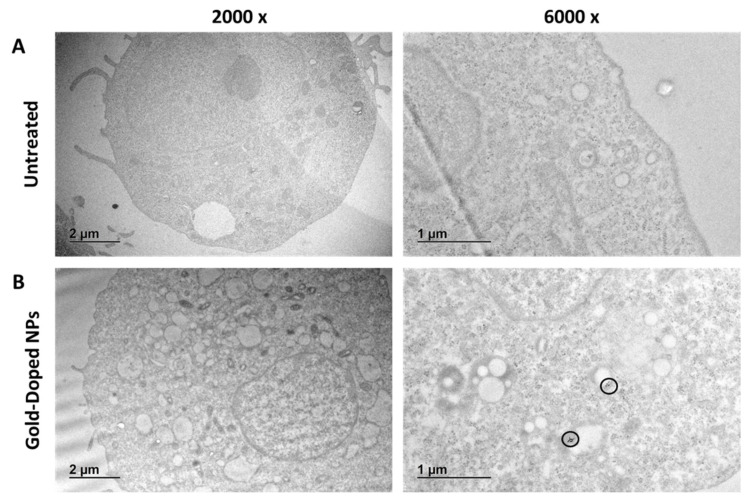
TEM analysis of U87-MG cells. (**A**) Sections of untreated U87-MG cells at two different magnifications. (**B**) Sections of U87-MG cells exposed for 24 h to gold-doped NPs (500 µg/mL) at two different magnifications. Electron-dense 5 nm gold NPs appear as black dots, highlighted by black circles.

**Figure 10:**
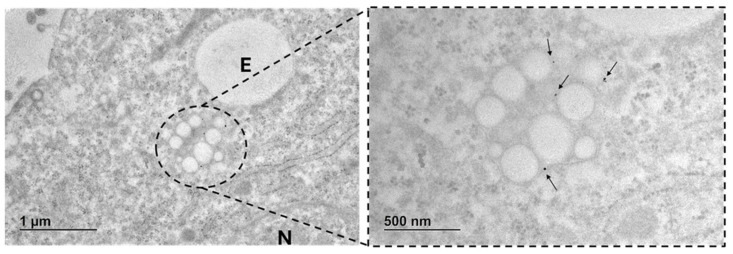
High magnification image of U87-MG cells exposed for 24 h to gold-doped NPs (500 µg/mL). NPs localized within endosomes (E) and near the nucleus (N). Gold NPs are indicated with black arrows in the higher magnification detail.

The cellular uptake and intracellular localization of gold-doped NPs were also investigated through TEM. Figure 9A shows the internal ultrastructure of untreated control cells, in which no lipid droplets are visible. On the other hand, cells incubated for 24 h with gold-doped NPs displayed multiple lipid droplet structures inside the cytoplasm and endosome pathways (Figure 9B). Several electron-dense inclusions, appearing as dark dots (Figure 9B), were detected. These darker areas were associated with the gold-dopant tracer in NPs and confirmed the successful internalization of gold-doped NPs and their localization within intracellular structures. Indeed, high magnification images (Figure 10) showed that gold-doped NPs were predominantly confined within endosomal compartments near the nucleus, suggesting endo-phagocytosis uptake [91,92].

Moreover, the uptake of the gold-doped NPs did not compromise the intracellular morphology of U87-MG cells, as the cytoplasmic organelles, including the nucleus and mitochondria, appeared intact, with a similar shape and structure as observed in the untreated control cells [93].

## 4. Discussion

Nanomedicines have been demonstrated to substantially improve drug delivery by mitigating side effects, enhancing drug accumulation at the target site, and improving cellular internalization [4,6]. Despite these transport advantages, the precise spatiotemporal localization and real-time monitoring of NPs within living systems are still limited, particularly for polymer-based nanomedicines, which are hard to discriminate from other biological compartments. These tracking limitations hinder the comprehensive understanding of the fate and biological impact of polymer NPs [17,18], limiting their potential translation. Traditional fluorescence-based methods, such as flow cytometry and fluorescence microscopy, suffer from fluorophore instability and resolution constraints that impede the detection of individual particles [32]. Similarly, advanced techniques based on electron microscopy and spectroscopy, albeit extremely sensitive, are incompatible with real-time analysis and have been applied almost exclusively to metallic NPs [35,94].

Recent reports have highlighted the potential of combining multimodal detection methods to enable a more comprehensive assessment of the interactions of NPs with living cells. For example, Andrian et al. employed a combined light and electron microscopy approach to precisely localize fluorescently labelled polymeric NPs inside cellular compartments via direct stochastic optical reconstruction microscopy (dSTORM) correlated with TEM images [95]. The dSTORM and TEM images were separately acquired on each sample and then overlaid by relying on fiducial markers visible in both images. This correlation method allowed the visualization of cellular ultrastructure via TEM with the simultaneous localization of NPs, which were not directly visible under TEM. However, manual registration of multiple fiducial points remains time-consuming and operator-dependent, limiting accuracy and high-throughput.

Similarly, Gilleron et al. [92] monitored the in vitro uptake of lipid NPs containing labelled small interfering RNAs (siRNAs). SiRNA was labelled with either a fluorophore for fluorescence microscopy detection or gold NPs for electron microscopy visualization. This approach enabled monitoring liposome trafficking across intracellular compartments via immunofluorescence and cell ultrastructure visualization under TEM. While this approach facilitated analysis of cargo internalisation, the two labelling methods were not integrated within a single platform, thereby limiting simultaneous detection using combined techniques. Furthermore, this method was restricted to siRNA-based systems without allowing tracking of the carrier itself, as the monitoring depended on labelling the cargo. Designing a similar multimodal platform that integrates both tracking approaches into a single carrier would significantly enhance its generalization for the delivery of different therapeutic agents.

Herein, we implemented a metal-doping strategy to facilitate tracking of polymer NPs via multimodal imaging modalities, without the need for fluorescence labelling. To this aim, we successfully obtained gold-doped polymer/lipid NPs via a simple nanoprecipitation method and demonstrated their tracking via TEM and holotomography imaging [43].

We successfully produced stable, small-sized, gold-doped NPs. TEM imaging demonstrated that gold NPs were well distributed across the particle volume, localising within the polymer core and adjacent to the lipid shell. UV/Visible spectroscopy confirmed a loading efficiency of approximately 50%, comparable to results obtained in similar studies employing polymer [60,96] and lipid NPs [61,62].

The TEM analysis shows a random clustering of the 5 nm gold NPs within the polymeric core. This clustering phenomenon is known to cause the broadening and the redshift of the SPR peak in comparison to un-clustered nanoparticles, as also observed in this work [73]. This behaviour may be beneficial for potential applications in hyperthermal treatment [97]. Prior studies reported that the clustering of ultrasmall gold NPs within polymer cores results in a widening of the absorption spectrum, in turn causing a more pronounced temperature increase [54]. Future work may evaluate the photothermal properties of the platform to validate its potential as a theranostic tool integrating bioimaging, hyperthermia, and controlled drug delivery within a single particle. We showed that the gold doping process did not alter the main physicochemical properties of the NPs. Indeed, no significant variations in size or PDI were detected, suggesting that the NPs preserved their monodisperse nature after the doping process, in accordance with previous literature [98,99]. The incorporation of gold NPs resulted in a modest increase in the NP diameter, quantified at approximately 20 nm. While many prior studies have reported no changes in diameter following the incorporation of gold-based dopant in polymeric carriers [38,92], some works have noted a similar increment of 20–40 nm in size for lipid NPs [97,100,101]. This size increase may be considered a useful physical marker of successful encapsulation of the gold dopant within the polymeric shell [9,63]. The obtained size window, with approximately 90% of the particles presenting diameters in the range of 50–250 nm, has been shown to favour circulation, as well as to reduce hepatic and splenic filtration of NPs. Moreover, this size is compatible with passive tumour targeting via the enhanced permeability and retention (EPR) effect, making these formulations suitable for passive targeting of tumours [102,103,104,105,106]. Gold doping modified the surface properties of the carrier, resulting in a significant increase in the zeta potential from −47 ± 5 mV to −28 ± 4 mV. This shift toward a less negative surface charge was attributed to the nearly neutral charge of bare gold NPs and is consistent with previous literature, documenting changes in potential following the incorporation of gold particles [99,107]. Gold-doped NPs maintained their stability over 10 days under storage and physiological temperature, remaining monodispersed with minimal size variation (<10%) and only modest fluctuations in zeta potential compared to the undoped counterpart. These results confirmed that incorporation of gold NPs in the polymer/lipid NPs did not compromise their monodispersed nature nor their long-term physicochemical stability.

The hybrid nanoplatform is expected to exhibit prolonged systemic circulation and enhanced tumour accumulation, consistent with previous in vivo studies utilizing similar core–shell structures [12,50]. Our results demonstrate the stability of the gold-doped NPs over time, both in water and cell culture medium. Therefore, we expect minimal undesired release of the dopant in vivo and no negative impact on the circulation time and transport characteristics of the platform.

Furthermore, flow cytometry analyses quantitatively confirmed a rapid and efficient internalization of gold-doped NPs by glioblastoma cells and microglia, indicating that the doping procedure did not impair effective uptake of NPs by target cells. Indeed, the results were comparable to the uptake of undoped NPs with similar trends. For instance, a lower internalization was detected for microglia in comparison to tumour cells, which was attributed to the low activation state of the cells in this study [108,109,110]. Therefore, while the gold-doping procedure led to an alteration in surface charge, it did not affect cellular uptake, granting internalisation kinetics comparable to those observed with undoped NPs.

These findings validate the suitability of the gold-doped NP platform for non-invasive monitoring of internalisation kinetics via multimodal imaging, as the doping strategy did not alter cellular trafficking pathways or impede intracellular drug delivery [52].

Despite the small size of the metallic dopant, the results demonstrated a significant alteration of the optical properties (refractive index and absorbance) of the gold-doped NPs, ensuring their detectability through UV/Visible spectroscopy, TEM, and holotomography.

Holotomography leverages the distinct optical properties (RI) of different cell compartments, allowing their visualization without additional fluorescence counterstaining [44]. This technique has emerged in recent years for label-free live imaging of cells. While holotomography has been used to track metallic NPs [45,49], its application to polymer and lipid carriers has been restricted by the similarity between the optical properties of the particles and those of biological structures. This study demonstrated that the alteration of the refractive index after gold doping successfully enhanced the optical contrast, allowing polymer NPs to be discriminated from cell compartments using holotomography imaging. Following administration of gold-doped NPs, distinct high-RI areas were observed within the cell cytoplasm compared to untreated controls, with the number and spatial extension of these regions increasing proportionally with NP concentration. Correlative imaging combining holotomography and confocal microscopy confirmed a direct spatial correlation between these high-RI spots and the fluorescence signals from rhodamine-labelled gold-doped NPs, indicating that the high-RI areas accurately represent the intracellular accumulation of the gold-doped NPs. These findings demonstrated that gold doping can be effectively leveraged for label-free visualization of the intracellular accumulation of NPs via holotomography, offering a less intrusive and real-time alternative to traditional fluorescence-based methods [41,45]. The high colocalization between the fluorescent label on the NPs and the high RI strongly suggests that gold-doped NPs can be identified as the regions of high RI. It must be noted that cell structures of intrinsically high RI, such as lipid droplets, may provide false results when fluorescent labelling is not employed. Although our results and previous studies [45,88] suggest that the influence of these artifacts is limited, future studies may explore a combination of holotomography with other label-free techniques, such as Raman spectroscopy, to more precisely identify artifacts and establish robust RI segmentation thresholds.

Holotomography offers rich quantitative capabilities beyond simple RI mapping, providing detailed metrics to evaluate NP aggregation, accumulation, and overall intracellular distribution [111]. While the full potential of this powerful technique is not fully explored in the current work, the inherent utility of our gold-doped platform can be maximized through subsequent methodological development. Achieving truly rigorous analysis of intracellular NP localization inside different cellular compartments demands the development of machine learning and advanced image processing pipelines [112]. Such automated systems would enable efficient segmentation and feature extraction, ultimately complementing or even replacing analogue evaluations performed with fluorescence immunostainings and TEM imaging.

Furthermore, the presence of the high electron-dense gold tracers in our gold-doped NPs enabled their visualization and the assessment of their localization within intracellular compartments under TEM. The direct detection of organic NPs through TEM has been hampered by their low electron density and limited contrast from cell components [25]. Here, cells incubated with gold-doped NPs displayed multiple structures featuring electron-dense inclusions associated with the gold tracer, while these inclusions were absent in untreated controls. These data confirmed successful internalization of gold-doped NPs, and their localization was precisely identified within endosomal compartments near the nucleus.

Future analysis employing correlative light and electron microscopy (CLEM) could be beneficial to further validate the reliability of this detection mechanism, allowing for the definitive verification of co-localization between the metallic dopants and fluorescently labelled NPs.

Our results underscore the potential of multimodal nanoplatforms to provide a more comprehensive and accurate understanding of NPs’ interactions with biological systems through multiple complementary techniques. Moreover, this approach could be extended to other analytical methodologies. For instance, the doping with 5 nm gold NPs may enable the detection of polymeric nanocarriers in vitro and in vivo through highly efficient quantification techniques, such as single-particle inductively coupled plasma mass spectrometry (sp ICP-MS), which is currently not applied to polymeric materials due to the ubiquitous presence of similar carbon-based compounds in biological matrices [17,113]. Recent investigations demonstrated that doping nano plastics with ultrasmall gold NPs enabled the study of their uptake, both in vitro and in vivo, through single-cell ICP-MS (sc ICP-MS) [56,114], paving the way for even more complex applications of the gold-doped platform. For instance, the quantification of intracellular gold content via ICP-MS may provide a sensitive, indirect measure of NP internalization, capitalizing on the attogram-level absolute limit of detection for gold [56]. Furthermore, this analysis could be complemented by flow cytometry [115], combining the high elemental sensitivity of the former technique with the superior cell counting and population identification accuracy of the latter [116,117], to provide a more reliable quantification of intracellular NPs [118,119]. The proposed gold-doping approach can be potentially exploited in future investigations to allow elemental analysis techniques such as ICP-MS, which are typically incompatible with organic carriers, to achieve quantitative biodistribution or environmental tracing of polymeric nanocarriers.

While gold nanoparticles were chosen for their superior biocompatibility over metals like copper or manganese and their potential for combined imaging and therapeutic applications [43,120], this doping methodology can be readily extended to other metallic tracers with similar theranostic properties. For instance, silver nanoparticles could offer enhanced detection via TEM and holotomography while conferring antibacterial properties [121,122], while iron oxide nanoparticles may allow combined contrast-enhancement capabilities with hyperthermia [49,123].

## 5. Conclusions

In this study, we successfully produced gold-doped polymer NPs via a reproducible and straightforward nanoprecipitation method, yielding nanocarriers with optimal long-term stability and size compatible with therapeutic applications. We demonstrated that holotomography microscopy can be employed for real-time, label-free monitoring of gold-doped NP uptake, with a strong correlation observed between high-RI regions and fluorescence signals. Moreover, the doping procedure enabled NP visualization inside intracellular compartments through TEM imaging, achieving high-resolution monitoring of NP uptake and trafficking. These findings suggest that the proposed gold-doped NPs may serve as versatile nanoplatforms compatible with multimodal imaging and analytical techniques, to enable a more accurate understanding of nanocarrier behaviour within biological systems.

## Figures and Tables

**Table 1:** Summary table of undoped and gold-doped NP properties. * *p* < 0.05, ** *p* < 0.01, *** *p* < 0.01, **** *p* < 0.0001. N/A: not applicable.

	Undoped NPs	Gold-Doped NPs	*p* Value
**Size (nm) (DLS)**	120 ± 8	140 ± 13	0.006 (**)
**Size (nm) (TEM)**	124 ± 43	141 ± 52.8	<0.0001 (****)
**Size (nm) (NTA)**	101 ± 2	124 ± 1	0.0004 (***)
**PDI (%)**	12 ± 3	11 ± 6	0.9961
**Z potential (mV)**	−47 ± 5	−28 ± 4	<0.0001 (****)
**Density (NPs/mL)**	3.3 ± 0.1 × 10^12^	4.0 ± 0.2 × 10^12^	0.01 (*)
**Gold-doping** **efficiency (%)**	N/A	56.3 ± 7.6% (indirect)	N/A
	N/A	47.7 ± 2.6% (direct)	N/A

**Table 2:** Descriptive parameters derived from quantitative image analysis of U87-MG cells following 2-h incubation with rhodamine-labelled gold-doped NPs (500 μg/mL). Data were collected from holotomography images encompassing 230 individual cells identified across 20 distinct fields of view.

**Colocalization between fluorescent and high-RI voxels**	92 ± 3%
**Average Aggregate Size**	0.55 ± 0.50 µm^3^
**Average volume (%) occupied by NPs**	6.7 ± 5.5%
**Average number of aggregates per cell**	50 ± 45

## Data Availability

The original contributions presented in this study are included in the article. Further inquiries can be directed to the corresponding author.

## Supplementary Materials

The following supporting information can be downloaded at: https://www.mdpi.com/article/10.3390/pharmaceutics17121612/s1: Table S1. Characteristic parameters of the 5 nm gold NPs. Core size, particle density, and absorption peak (highlighted with the asterisk, *) were provided by the producer, while the hydrodynamic diameter, zeta potential and PDI were measured by DLS analysis. Figure S1. Expected structure of the undoped and gold-doped NPs. The outer lipid shell features three key components: DSPE-PEG for extended circulation and stability; Egg-PG for lipid shell integrity; and Egg-Liss-Rhod PE for fluorescence detection. Created in BioRender. Mattu, C. (2025) https://BioRender.com/0i3efwp. Figure S2. Mean diameter distributions for (**A**) undoped and (**B**) gold-doped NPs based on manual measurements of at least 400 particles from TEM images. Figure S3. Stability curve, showing multiple measurements of the PDI (*n* = 3) of gold-doped NPs over 10 days of incubation in ultra-pure water at 4 °C (continuous line) and 37 °C (dotted line). Figure S4. Hydrodynamic diameter (**A**), polydispersity index (PDI) (**B**), and Z-potential (**C**) of undoped NPs and gold-doped NPs measured over time in culture medium at 37 °C (*n* = 3). Statistical analysis was performed by Ordinary Two-way ANOVA, comparing the different timepoints at which each group was observed. * *p* < 0.05, ** *p* < 0.01, *** *p* < 0.001, **** *p* < 0.0001. Figure S5. Cell viability assay after incubation of (**A**) human microglia (HMC3) and (**B**) human astrocytes (HASTR/ci37) with gold-doped NPs at different concentrations for 24 h, 48 h, and 72 h (*n* = 3). Figure S6. (**A**) Representative confocal images of rhodamine-labelled undoped NPs internalized by HMC3 after 24 h of incubation with a concentration of 500 μg/mL of NPs. F-actin was stained with phalloidin (green), nuclei were stained with DAPI (blue), and NPs were visualized in red. Representative flow cytometry plots for HMC3 cells after 2 h (**B**) and 24 h (**C**) of incubation with different concentrations of rhodamine-labelled gold-doped NPs. Flow cytometry quantification of the percentage of NP-positive cells (**D**) and of mean fluorescence intensity (**E**) on HMC3 cells after 2 and 24 h of incubation with different concentrations of rhodamine-labelled gold-doped NPs (*n* = 4). Statistical analysis was performed by Ordinary Two-way ANOVA, comparing the groups treated with the same concentrations at the two timepoints. ** *p* < 0.01, **** *p* < 0.0001. Figure S7. (**A**) Representative confocal images of rhodamine-labelled undoped NPs internalized by HASTR/ci37 after 24 h of incubation with a concentration of 500 μg/mL of NPs. F-actin was stained with phalloidin (green), nuclei were stained with DAPI (blue), and NPs are visualized in red. Flow cytometry quantification of the percentage of NP-positive cells (**B**) and of mean fluorescence intensity (**C**) on HASTR/ci37 cells after 2 and 24 h of incubation with different concentrations of rhodamine-labelled gold-doped NPs (*n* = 4). Statistical analysis was performed by Ordinary Two-way ANOVA, comparing the groups treated with the same concentrations at the two timepoints. *** *p* < 0.001, **** *p* < 0.0001. Figure S8. (**A**,**B**) Representative flow cytometry plots for U87-MG cells after 2 (**A**) and 24 (**B**) h of incubation with different concentrations of rhodamine-labelled gold-doped NPs. (**C**) Flow cytometry quantification of the mean fluorescence intensity for U87-MG cells after 2 h and 24 h of incubation with different concentrations of rhodamine-labelled gold-doped NPs (*n* = 4). Statistical analysis was performed by Ordinary Two-way ANOVA, comparing the groups treated with the same concentrations at the two timepoints. **** *p* < 0.0001. Figure S9. Internalization kinetics of gold-doped NPs by U87-MG cells over 12 h, visualised by combining fluorescence microscopy and holotomography imaging. U87-MG cells were incubated with different concentrations of gold-doped NPs: (**A**) 100 μg/mL, (**B**) 200 μg/mL. Rhodamine-labelled gold-doped NPs are shown in red over grayscale holotomography images. Figure S10. (**A**) UV–vis absorption spectra of 5 nm gold NPs at different particle concentrations. (**B**) The relationship between the NPs concentration and the amplitude of the absorption peak at 518 nm. The distribution was modelled using a simple linear regression (red line) with a confidence interval of 95% (dotted lines). Figure S11. Average RI histograms (above) and representative RI holotomography images of (**A**) native U87-MG cells and cells incubated with gold-doped NPs at different concentrations: (**B**) 100 μg/mL, (**C**) 200 μg/mL, (**D**) 500 μg/mL.

## Abbreviations

The following abbreviations are used in this manuscript:

TEM	Transmission Electron Microscopy
NP	Nanoparticle
ICP-MS	Inductively Coupled Plasma Mass Spectrometry
EDS	Energy Dispersive X-Ray Spectroscopy
RI	Refractive Index
PU	Polyurethane
PCL	Poly(Ε-Caprolactone)
EGG-PG	L-A-Phosphatidylglycerol (Egg, Chicken) (Sodium Salt)
DSPE-PEG	1,2-Dioleoyl-Sn-Glycero-3-Phosphoethanolamine-N-[Methoxy(Polyethylene Glycol)-2000] (Ammonium Salt)
Egg-Liss-Rhod PE	L-A-Phosphatidylethanolamine-N- (Lissamine Rhodamine B Sulfonyl) Ammonium Salt
ACN	Acetonitrile
DLS	Dynamic Light Scattering
PDI	Polydispersity Index
NTA	Nanoparticle Tracking Analysis
LE	Loading Efficiency
DAPI	4′,6-Diamidino-2-Phenylindole, Dihydrochloride
SD	Standard Deviation
SPR	Surface Plasmonic Resonance
dSTORM	Direct Stochastic Optical Reconstruction Microscopy
EPR	Enhanced Permeability and Retention
CLEM	Correlative Light and Electron Microscopy
sp ICP-MS	Single-Particle ICP-MS
sc ICP-MS	Single-Cell ICP-MS
