# Supplementary material for: Gold-Doped Hybrid Nanoparticles: A Versatile Tool for Multimodal Imaging of Cell Trafficking
Source: Pharmaceutics. 2025 Dec 15;17(12):1612. doi: 10.3390/pharmaceutics17121612 (PMC12737188; doi:10.3390/pharmaceutics17121612)
Supplement: Supplementary file 1 [file pharmaceutics-17-01612-s001.zip › pharmaceutics-4028144-supplementary.pdf]

pharmaceutics

Supplementary Materials: Gold-Doped Hybrid Nanoparticles: A Versatile Tool for Multimodal Imaging of Cell Trafficking

Andrea Bezze, Jessica Ponti, Deborah Stanco, Carlotta Mattioda and Clara Mattu

Table S1. Characteristic parameters of the 5 nm gold NPs. Core size, particle density and absorption peak (highlighted with the asterisk, *) were provided by the producer, while the hydrodynamic diameter, zeta potential and PDI were measured by DLS analysis.

**Core size**	**5 ± 2 nm**	*
**Particle Density**	5.47×10^{13} particles/mL	*
**Absorbance peak**	518 nm	*
**Hydrodynamic diameter**	18.6 ± 0.4 nm	
**Zeta potential**	−2.3 ± 0.9 mV	
**PDI**	10 ± 1 %	

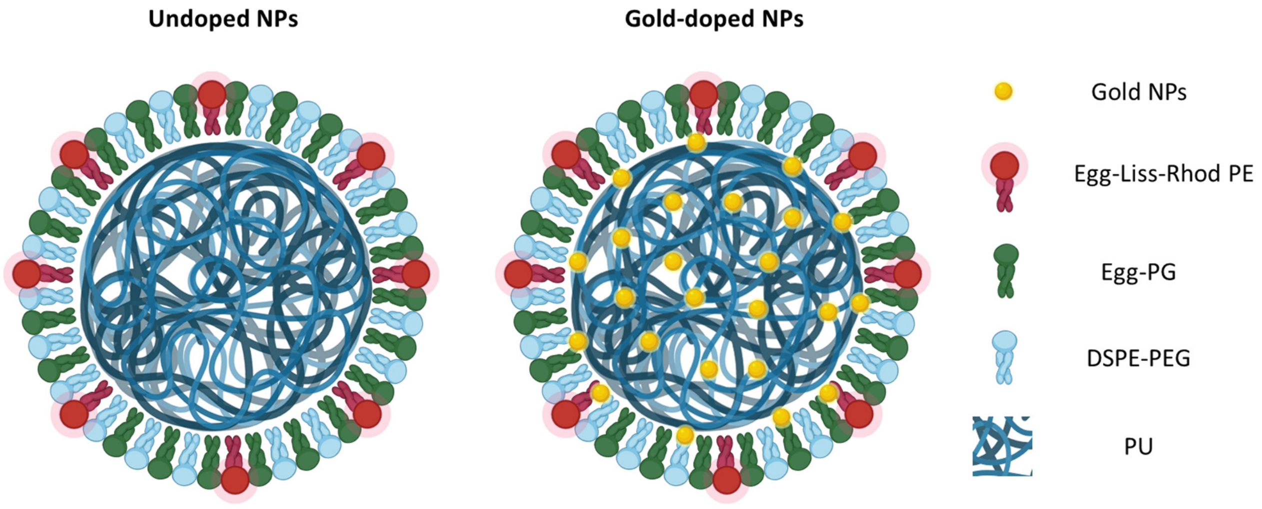

Figure S1. Expected structure of the undoped and gold-doped NPs. The outer lipid shell features three key components: DSPE-PEG for extended circulation and stability; Egg-PG for lipid shell integrity; and Egg-Liss-Rhod PE for fluorescence detection. Created in BioRender. Mattu, C. (2025) https://BioRender.com/0i3efwp.

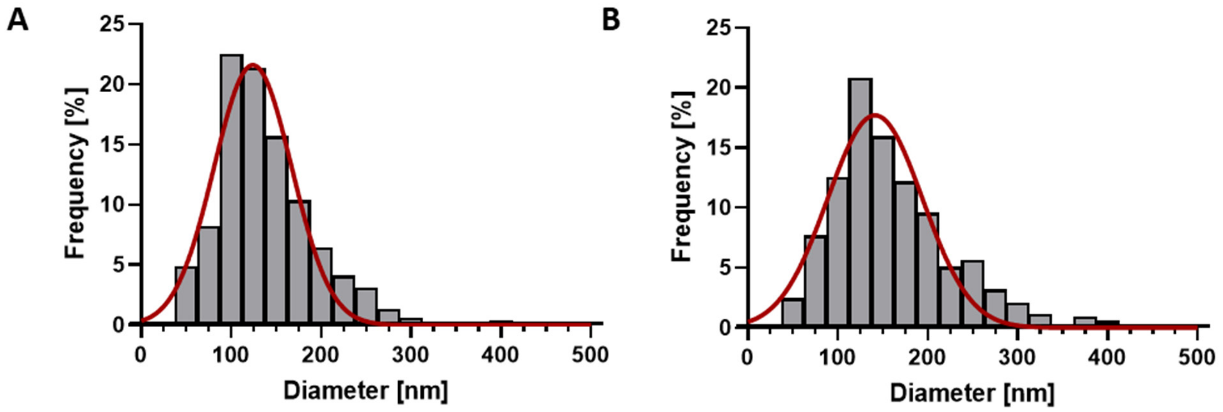

Figure S2. Mean diameter distributions for (**A**) undoped and (**B**) gold-doped NPs based on manual measurements of at least 400 particles from TEM images.

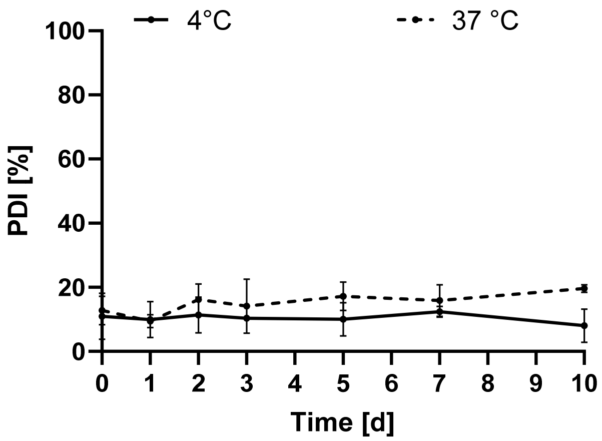

Figure S3. Stability curve, showing multiple measurements of the PDI (n = 3) of gold-doped NPs over 10 days of incubation in ultra-pure water at 4 °C (continuous line) and 37 °C (dotted line).

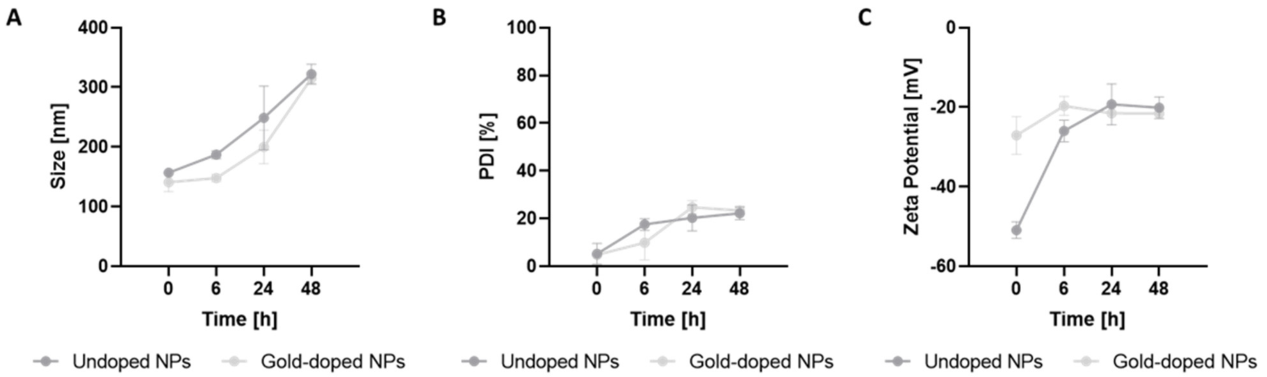

Figure S4. Hydrodynamic diameter (**A**), polydispersity index (PDI) (**B**), and Z-potential (**C**) of undoped NPs and gold-doped NPs measured over time in culture medium at 37 °C (n = 3). Statistical analysis was performed by Ordinary Two-way ANOVA, comparing the different time points at which each group was observed. *p<0.05, **p < 0.01,***p<0.001, ****p<0.0001.

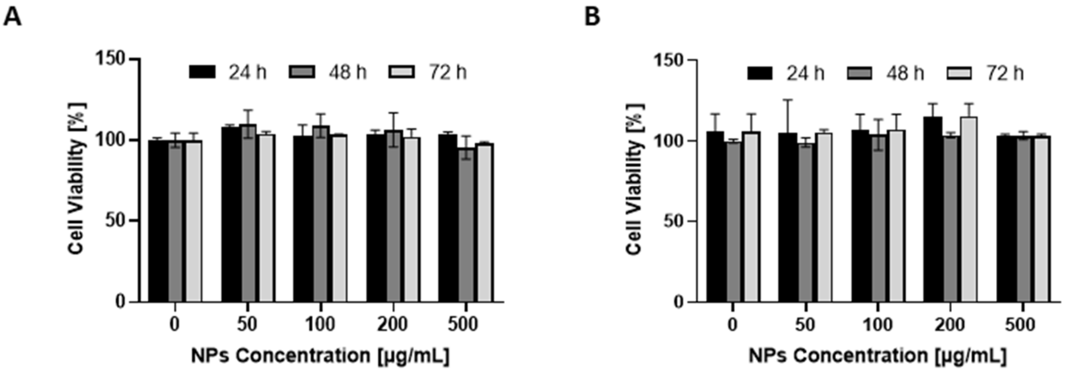

Figure S5. Cell viability assay after incubation of **A**) human microglia (HMC3) and **B**) human astrocytes (HASTR/ci37) with gold-doped NPs at different concentrations for 24 h, 48 h, and 72 h (n = 3).

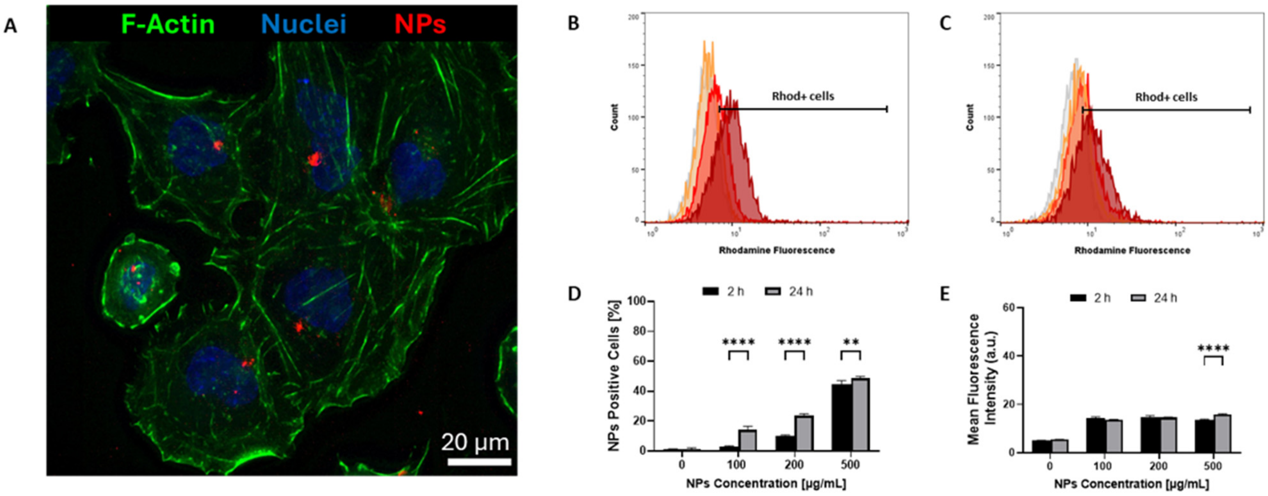

Figure S6. (**A**) Representative confocal images of rhodamine-labelled undoped NPs internalized by HMC3 after 24h of incubation with a concentration of 500 µg/mL of NPs. F-actin was stained with phalloidin (green), nuclei were stained with DAPI (blue), and NPs were visualized in red. Representative flow cytometry plots for HMC3 cells after 2 h (**B**) and 24h (**C**) of incubation with different concentrations of rhodamine-labelled gold-doped NPs. Flow cytometry quantification of the percentage of NPs-positive cells (**D**) and of mean fluorescence intensity (**E**) on HMC3 cells after 2 and 24 h of incubation with different concentrations of rhodamine-labelled gold-doped NPs (n = 4). Statistical analysis was performed by Ordinary Two-way ANOVA, comparing the groups treated with the same concentrations at the two timepoints. **p<0.01, ****p<0.0001.

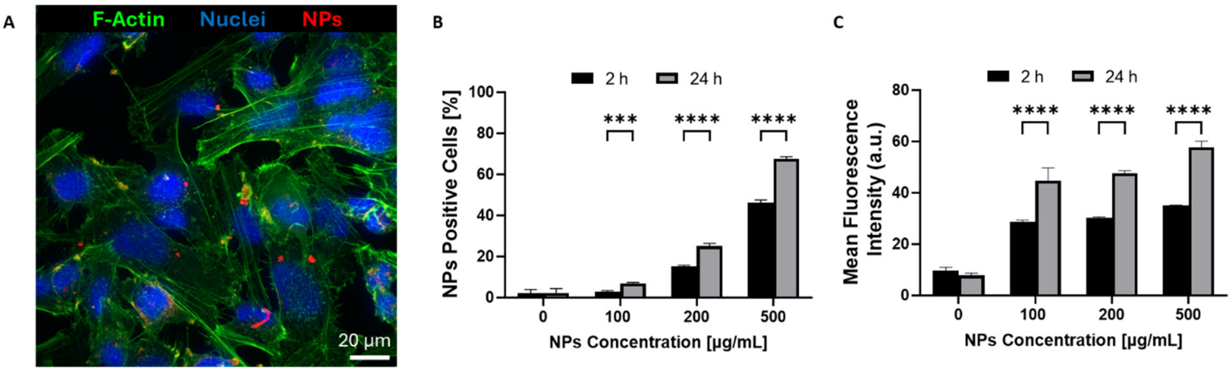

Figure S7. (**A**) Representative confocal images of rhodamine-labelled undoped NPs internalized by HASTR/ci37 after 24 h of incubation with a concentration of 500 µg/mL of NPs. F-actin was stained with phalloidin (green), nuclei were stained with DAPI (blue), and NPs were visualized in red. Flow cytometry quantification of the percentage of NPs-positive cells (**B**) and of mean fluorescence intensity (**C**) on HASTR/ci37 cells after 2 and 24 h of incubation with different concentrations of rhodamine-labelled gold-doped NPs (n = 4). Statistical analysis was performed by Ordinary Two-way ANOVA, comparing the groups treated with the same concentrations at the two timepoints. ***p<0.001, ****p<0.0001

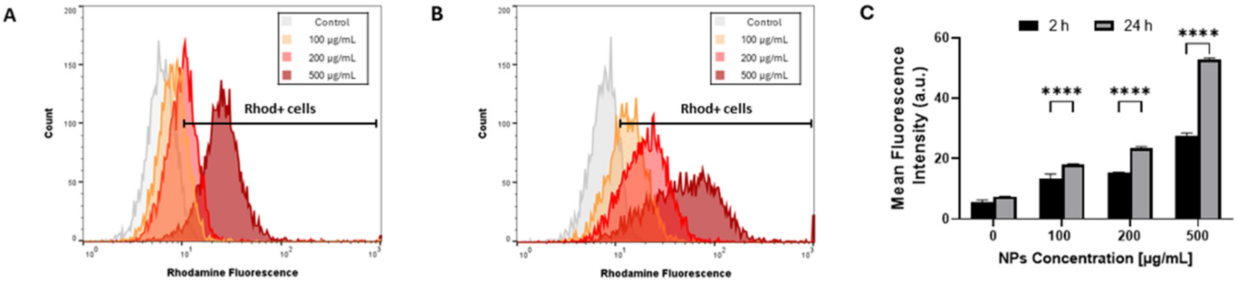

Figure S8. (**A**, **B**) Representative flow cytometry plots for U87-MG cells after 2 (**A**) and 24 (**B**) h of incubation with different concentrations of rhodamine-labelled gold-doped NPs. (**C**) Flow cytometry quantification of the mean fluorescence intensity for U87-MG cells after 2 h and 24 h of incubation with different concentrations of rhodamine-labelled gold-doped NPs (n = 4). Statistical analysis was performed by Ordinary Two-way ANOVA, comparing the groups treated with the same concentrations at the two timepoints. ****p<0.0001.

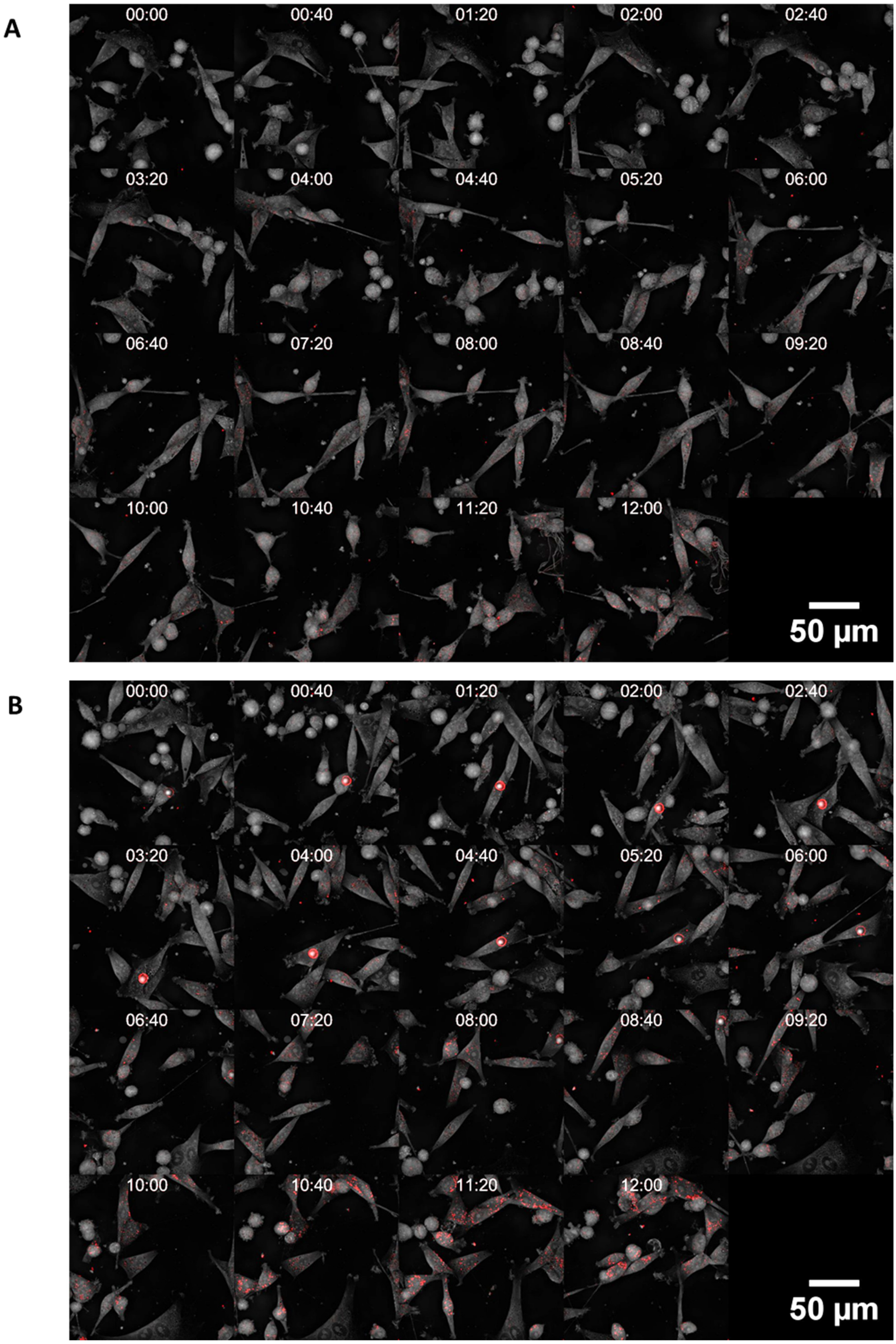

Figure S9. Internalization kinetics of gold-doped NPs by U87-MG cells over 12 h, visualised by combining fluorescence microscopy and holotomography imaging. U87-MG cells were incubated with different concentrations of gold-doped NPs: **A**) 100 µg/mL, **B**) 200 µg/mL. Rhodamine-labelled gold-doped NPs are shown in red over grayscale holotomography images.

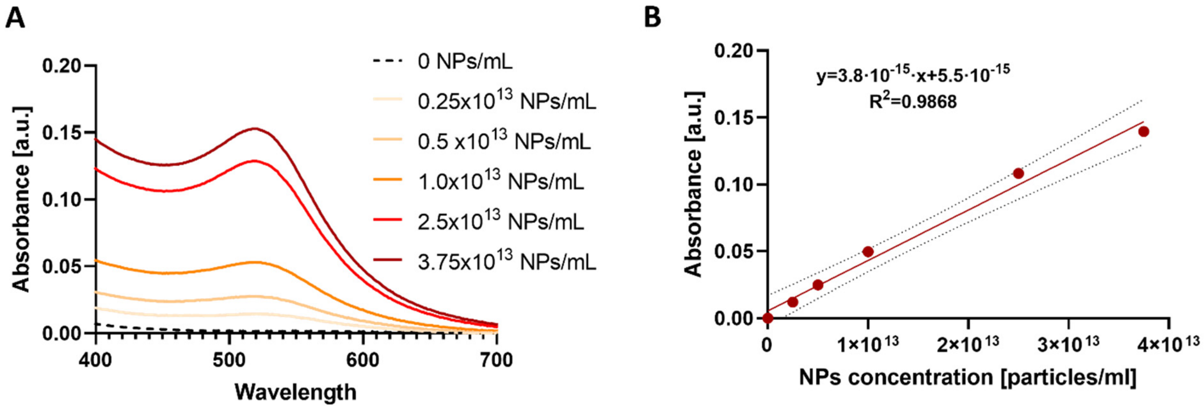

Figure S10. A) UV–vis absorption spectra of 5 nm gold NPs at different particle concentrations. **B**) The relationship between the NPs concentration and the amplitude of the absorption peak at 518 nm. The distribution was modelled using a simple linear regression (red line) with a confidence interval of 95% (dotted lines).

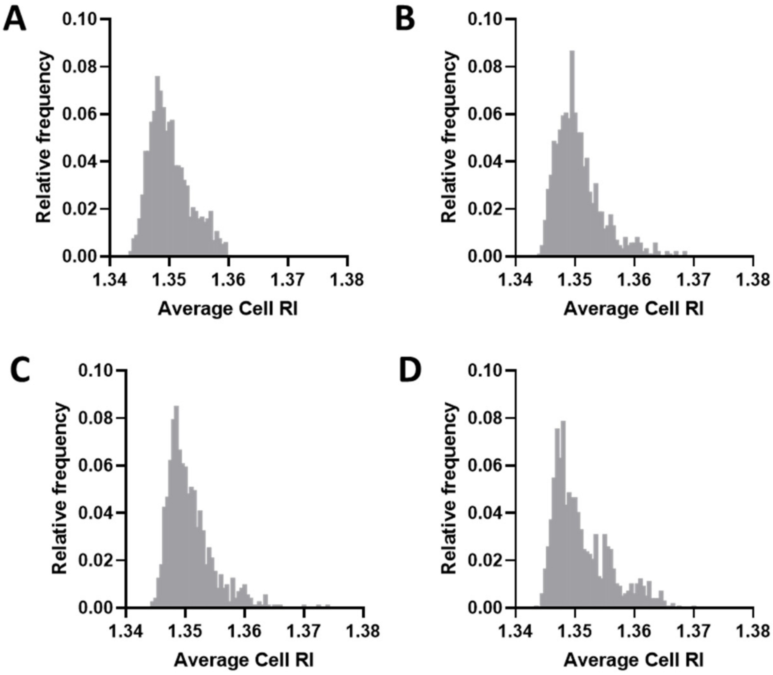

Figure S11. Average RI histograms (above) and representative RI holotomography images of **A**) native U87-MG cells and cells incubated with gold-doped NPs at different concentrations: **B**) 100 µg/mL, **C**) 200 µg/mL, **D**) 500 µg/mL.
